# Determination of arginine catabolism by salivary pellet^☆^

**DOI:** 10.1016/j.mex.2014.01.001

**Published:** 2014-01-22

**Authors:** M.A. Hoogenkamp, J.M. ten Cate

**Affiliations:** Academic Centre for Dentistry Amsterdam (ACTA), University of Amsterdam and Free University Amsterdam, Department of Preventive Dentistry, Gustav Mahlerlaan 3004, 1081 LA Amsterdam, The Netherlands

**Keywords:** Arginolytic activity assay in saliva, Arginine, Arginolytic, Ammonium, Salivary pellet, Oral bacteria

## Abstract

To determine the formation of ammonium from arginine by oral bacteria residing in saliva and dental plaque, an arginolytic activity assay based on the work described by Nascimento et al. [2] was developed. Following the original methodology, insufficient ammonium production could be determined.

To improve the method for our research goal, the following modifications were made to the original protocols:

• The following changes were made to the arginine catabolism assay resulting in a 1000-fold increase in sensitivity: (i) the salivary pellet was washed and concentrated five times resulting in the removal of low density compounds interfering with the assay, (ii) the pH of the Tris–maleate buffer was increased from 6.0 to 7.5 resulting in a better conversion of arginine to ammonium and (iii) the incubation time was increased to 3 h to ensure that non-responders and salivary pellets low in cell numbers could yield detectable levels of ammonium.
• Removal of a centrifuge step from the protein determination resulted in a higher protein yield improving the accuracy of the assay.
• Changing from the use of the toxic, environmentally hazardous, mercury containing Nessler's reagent to a colorimetric enzyme assay achieved a safer and greener determination of ammonium concentration.

## Graphical abstract

**Figure:**
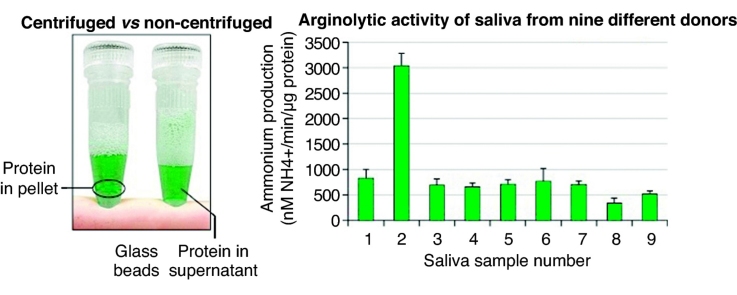

## Method details

In short, the assessment of the arginolytic potential involved the following steps: (i) preparation of the salivary pellet to concentrate the oral bacteria present in the saliva; (ii) performing the arginolytic activity assay to assess the arginine catabolism; (iii) determination of the amount of ammonium produced from arginine; and (iv) determination of the protein weight of the salivary pellet. The final concentrations of ammonium were corrected per time unit and protein weight.

### Preparation of the cell suspension

While optimizing the arginolytic activity assay, the pH of the Tris–maleate buffer was raised from pH 6.0 to pH 7.5. This was done as the arginolytic activity of a range of oral micro-organisms and the salivary pellet significantly produced more ammonium under pH 7.5 as compared to pH 6.0. The cell suspensions were prepared as follows:

1. Stimulated human saliva from various donors was thawed.
2. Four milliliter of each saliva sample was centrifuged for 10 min at 4000 × *g* at 4 °C.
3. The salivary pellet was washed once with 800 μl 10 mM Tris–maleate buffer, pH 7.5 (Sigma) to remove low density salivary components.
4. The pellet was re-suspended in a final volume of 800 μl Tris–maleate buffer.

### Arginolytic activity assay

In comparison to the original protocol the incubation time was lengthened to three hours. This was done to ensure that ammonium formation, in salivary pellets, low in cell amounts and saliva from non-responders, was still measurable.

To assess the arginolytic activity of the prepared cell suspensions, the following protocol was followed:

1. Of each cell suspension 237.5 μl was pipetted, in duplicate, into a non-skirted 96-well PCR plate (Corning) and pre-incubated at 37 °C in a thermocycler (Eppendorf).
2. The remaining cell suspension was stored at −20 °C for protein determination.
3. The arginolytic activity assay was started by adding 12.5 μl 1 M arginine to a final concentration of 50 mM (Sigma) to each cell suspension.
4. The plate with the cell suspensions was incubated at 37 °C for 3 h.
5. Immediately after the addition of the arginine, and after the three-hour incubation period, 50 μl samples were taken, transferred to a PCR plate (VWR) and placed on ice for 5 min.
6. After cooling, the samples were heat inactivated for 5 min at 80 °C, to stop all enzymatic reactions.
7. The PCR plate was centrifuged for 10 min at 1509 × *g* at 4 °C.
8. The supernatants were transferred to a microtiter plate (Greiner), the plate was sealed and stored at −20 °C until further analysis.

### Ammonium determination

To determine the amount of ammonium produced from the catalysis of arginine, an assay was used as based on the method of Da Fonseca et al. [1].

#### Preparation of the reagents

1. The reaction buffer (TK-buffer) containing 0.5 M triethanolamine and 15 mM α-ketoglutaric acid (both Sigma), pH 8.0 was prepared. This buffer was stored at 4 °C for maximum of 4 weeks.
2. Fresh NADH stock solution was prepared by dissolving 40 mg NADH (Roche) in 10 ml TK-buffer.
3. To create a NADH work solution, the NADH stock solution was further diluted (10 times) in TK-buffer. This NADH work solution was stored on ice until further use.
4. The GIDH stock solution was prepared by adding 3.6 ml MilliQ water to 3000 U glutamate dehydrogenase (Roche). This stock solution was stored at 4 °C.
5. Prior to the assay, a GIDH work solution was prepared by diluting the GIDH stock solution, 10 times in MilliQ water. The GIDH work solution was stored on ice until further use.
6. A 4 mM ammonium stock was prepared by diluting a 40 mM ammonium sulfate (NH_4_)_2_SO_4_ (Sigma), 20 times in TK-buffer.
7. Ammonium standards were prepared in the range of 0–2.8 mM, with 0.4 mM intervals.

#### Ammonium assay

1. A 96-well microtiter plate (Greiner) was filled with 120 μl MilliQ water per well.
2. 65 μl of NADH work solution was added to each well.
3. 10 μl samples (obtained from the arginolytic activity assay) were added in duplicate, to the appropriate wells.
4. 10 μl of the eight standards was added in duplicate to the appropriate wells.
5. The plate was mixed for 5 min on a microtiter plate shaker.
6. The absorbance *A*1 (*λ* = 340 nm) was read.
7. The enzymatic reaction was started by adding 20 μl GIDH working solution to each well.
8. The plate was mixed for 30 s on a microtiter plate shaker.
9. The plate was incubated for 20 min at 20–25 °C.
10. The absorbance *A*2 (*λ* = 340 nm) was read.

**Fig. 1:**
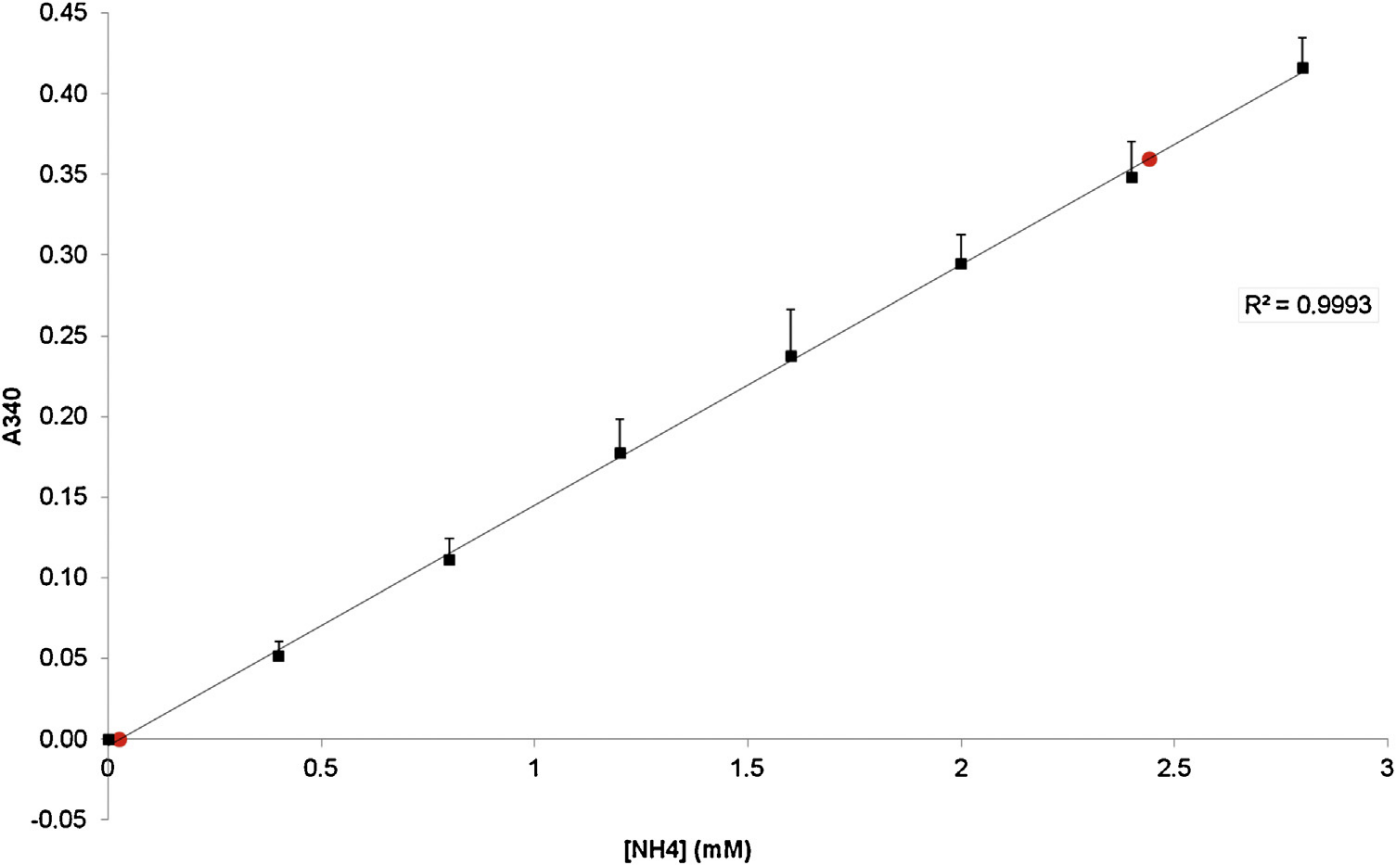
The standard curve for the ammonium determination. Each black square represents the average absorbance (340 nm) of four individual standard curves determined in duplo. Also shown are the correlation coefficient and the lower and upper detection limits, depicted with the red dots.

The Δ*A* (*A*1 − *A*2) was calculated and the values extrapolated against the ammonium standards. The calibration curve for the ammonium assay was linear up to 3.5 mM ammonium. The lower detection limit was defined as the average absorbance of the lowest concentration measured plus three times the standard deviation. The upper detection limit was defined as the average absorbance of the highest concentration measured minus three times the standard deviation. Fig. 1 shows an example of the reproducibility and accuracy of the standard curve including the upper and lower detection limit.

The obtained ammonium values were corrected for time and protein mass. To illustrate the sensitivity of the method, the Graphical Abstract shows the arginolytic activity of saliva from nine different donors, expressed as the amount of ammonium (nM) produced per minute per μg protein.

### Protein determination of the salivary pellet

The protein concentration of the cell suspensions used in the arginolytic activity assay was determined to normalize the ammonium formation by the salivary pellets with regards to differences in biomass.

In the original protocol [2], a centrifuge step had to be performed after the bead beating steps. Unfortunately, by centrifuging the samples, the cell walls were pelleted on top of the glass beads (see Graphical Abstract). As a result, less protein is present in the supernatant. This subsequently leads to an underestimation of the protein concentration, as the supernatant is used for the protein determination according to Bradford. Hence this centrifugation step was omitted from the method.

To perform the modified protein determination the following steps were taken:

1. A 2 ml 96-deepwell plate (Greiner) was filled with 200 μl MilliQ water washed Ø = 0.1 mm glass beads (Biospec products) per well.
2. The samples from stored at step 2 of the arginolytic activity assay were thawed.
3. 100 μl of each sample was pipetted in duplicate, into the deepwell plate.
4. The plate was sealed with a silicone mat.
5. The plate was subjected to two bead-beat cycles comprising of 30 s bead-beating, using a mini-Beadbeater 96 (Biospec products).
6. After each bead-beat step, the samples were cooled for 5 min on ice to prevent heating of the sample.
7. The deepwell plate was placed on a microtiter plate shaker (Labocat), and vortexed for 1 min.
8. The plate was taken off the shaker and the glass beads were left to settle for 5 min.
9. A 75 μl sample of the supernatant above the glass beads was taken.
10. The samples were transferred to a 96-well microtiter plate (Greiner).
11. The microtiter plate was placed on the microtiter plate shaker to center any residual glass beads.

Determine the protein concentration of the supernatant, in duplicate, according to the method of Bradford [3].

12. Each well of a 96-well microtiter plate (Greiner) was filled with 185 μl MilliQ water and 50 μl Bio-Rad Protein assay reagent.
13. Standards were prepared using bovine serum albumin (Sigma).
14. 15 μl of supernatant from step 11 was added to the reaction mixtures.
15. The samples were mixed vigorously by pipetting and 100 μl of the mixtures was transferred to a new 96-well microtiter plate.
16. The absorbance (*λ* = 595 nm) was determined and the protein concentration calculated.
